# Mechanisms of individual differences in heterotopic noxious analgesia (DNIC), an fMRI study

**DOI:** 10.1186/1129-2377-14-S1-P94

**Published:** 2013-02-21

**Authors:** A Vigàno, VB Bogdanov, Q Noirhomme, N Guy, R Dallel, S Laureys, C Phillips, J Schoenen

**Affiliations:** 1 University of Rome La Sapienza, Italy; 2 University of Utah, USA; 3 University of Liège, Belgium; 4 Université d'Auvergne., France

## Introduction

Pain responses can be suppressed by heterotopic continuous noxious conditioning, e.g. continuous noxious cold stimulation.

## Objective

These diffuse noxious inhibitory controls (DNIC) may be abnormal in migraine ( Sandrini et al 2006 ). DNIC effects are modulated by a number of prefrontal cortical areas ( Piché et al 2009 ) that could be dysfunctioning in migraine interictally.

## Methods

We examined in healthy volunteers blood oxygenation level dependent (BOLD) responses in prefrontal cortex to repeated continuous noxious cold stimulation. The relationships between those responses and degree of inhibition of laser-induced pain during heterotopic cold stimulation were analyzed.

## Results

Our results show that cold-induced BOLD response in anterior cingulate, orbitofrontal and lateral prefrontal cortices predict cold-induced heterotopic analgesia and attenuation of cerebral BOLD responses to laser stimulation. Prefrontal responses to the onset of cold stimulation were strongly related to the subsequent DNIC effect.

## Conclusion

We conclude that early responses to noxious conditioning are important for prediction of the analgesic DNIC effect. We hypothesize that this predictive effect of frontal cortices may be abnormal in chronic migraine.
